# Isolation and Characterization of *Lactobacillus paracasei* 85 and *Lactobacillus buchneri* 93 to Absorb and Biotransform Zearalenone

**DOI:** 10.3390/toxics10110680

**Published:** 2022-11-10

**Authors:** Min Gan, Jian Hu, Kai Wan, Xiangxiang Liu, Peirong Chen, Rui Zeng, Fuhua Wang, Yarong Zhao

**Affiliations:** 1 Institute of Quality Standard and Monitoring Technology for Agro-Products of Guangdong Academy of Agricultural Sciences, Guangzhou 510640, China; 2 Guangdong Provincial Key Laboratory of Quality & Safety Risk Assessment for Agro-Products, Guangzhou 510640, China; 3 Key Laboratory of Testing and Evaluation for Agro-Product Safety and Quality, Ministry of Agriculture and Rural Affairs, Guangzhou 510640, China; 4 Tangshan Food and Drug Comprehensive Testing Center, Tangshan 063000, China

**Keywords:** lactic acid bacteria, ZEN, absorption, biotransformation

## Abstract

As one of the most prevalent estrogenic mycotoxins in cereals and animal feed, zearalenone (ZEN) can cause serious reproductive disorders. ZEN control in food and feed commodities has been an imperative area of research. In this study, 87 lactic acid bacteria (LAB) were isolated from pickles and their ZEN (5 mg/L) removal abilities ranged from 0% to 68.4%. Then, five strains with potent ZEN removal ability (>50%) were identified: *Lactobacillus plantarum* 22, *L. plantarum* 37, *L. plantarum* 47, *L. paracasei* 85, and *L. buchneri* 93. Under optimization conditions (48 h, pH 4.0, 37 °C, and 5 mg/L), the highest ZEN removal abilities of *L. paracasei* 85 and *L. buchneri* 93 reached 77.7% and 72.8%, respectively. Moreover, the two lactic acid bacteria decreased the toxicity of ZEN, because the levels of β-zearalenol (β-ZOL) transformed from ZEN were more than two-fold higher than α-zearalenol (α-ZOL). Additionally, cell free supernatant and pellet biotransformation of ZEN to α-ZOL and β-ZOL in LAB were detected for the first time. Furthermore, chemical and enzymatical treatments combined with Fourier-transform infrared spectroscopy analysis indicated that exopolysaccharides, proteins, and lipids on the cell wall could bond to ZEN through hydrophobic interactions. Scanning electron microscopy indicated that cell structure damage occurred during the ZEN clearance to *L. buchneri* 93, but it did not with *L. paracasei* 85. In addition, various organic acids, alcohols, and esters of the two LAB participated in ZEN removal. Hence, *L. paracasei* 85 and *L. buchneri* 93 can be considered as potential detoxification agents for ZEN removal for food and feedstuff.

## 1. Introduction

Mycotoxins are toxic secondary metabolites produced by certain molds (fungi), nowadays, over 400 compounds have been identified [1]. Considering the outbreak frequency and toxic properties, scientists have focused on zearalenone (ZEN), aflatoxins (AFs), deoxynivalenol (DON), fumonisins (FBs), ochratoxin A (OTA), and patulin (PAT). These mycotoxins induce complicated diseases in animals and humans and threaten the safety of agricultural foodstuffs and animal feed. ZEN is a nonsteroidal estrogenic mycotoxin, produced by the *Fusarium* genus, including the species *Fusarium culmorum*, *Fusarium roseum*, *Fusarium graminearum*, and *Fusarium semitectum* [2]. The structure of ZEN is similar to natural estrogen, and it competitively binds to the estrogen receptors, resulting in reproductive toxicity, hepatotoxicity, nephrotoxicity, genotoxicity, and immune disturbance [3,4,5]. ZEN contamination is widespread. According to a previous survey, 70 of 117 cereal samples were positive for ZEN contamination, and 24 samples exceeded the highest permissible limit [6]. In 200 maize silage samples from China, ZEN was detected in 79.5% of the samples, and the highest concentration was 830 µg/kg, which exceeds the Chinese regulatory limit level [7].

ZEN is thermostable and cannot be decomposed easily during standard cooking procedures. Only 37% to 69% of ZEN could be decomposed after 30 to 60 min processing at 200 °C [8,9]. Although the daily exposure dose is relatively high, for infants (<12 months old), the mean dietary exposure estimates range from 3.3 to 88 ng/kg b.w. per day. Additionally, for the children and adolescents (≥1 to <18 years old), the highest exposure estimates range from 9.3 to 100 ng/kg b.w. per day [10]. Therefore, the control and elimination of ZEN in food and feed commodities is imperative. Numerous physical and chemical strategies have been developed to control the occurrence of ZEN contamination. Nevertheless, these strategies are limited due to the losses of important nutrients and high machinery costs. Biological detoxification is a promising strategy for ZEN elimination because of its advantages such as high efficiency, low cost, and energy savings. Various bacteria have been used to remove ZEN contamination, such as animal intestine bacteria *Clostridium sporogenes* F39, which has a ZEN degradation rate of 87.35% in 48 h [11]. Microbial ZEN degrading enzymes represent a feasible method for ZEN detoxification. Kakeya et al. reported that the mycoparasitic fungus *Clonostachys rosea* produced zearalenone lactonase to convert ZEN into a non-estrogenic compound [12]. *Gliocladium roseum* showed the ability to cleave the lactone ring of ZEN using lactonohydrolase enzyme [13]. Some of these methods, in fact, are impractical due to the risk of creating toxic residues through the production of unknown metabolites, or affecting the nutritional value and taste of foodstuffs. Thus, there are currently no legal regulations regarding the ZEN decontamination of food with fungi and related enzymes in the world.

Probiotics are defined by the FAO/WHO (2002) as live microorganisms that, when administered in adequate amounts could confer a health benefit on the host [14]. Common probiotics include *Lactobacillus*, *Bifidobacterium*, *Saccharomyces*, *Enterococcus*, and *Bacillus* [15]. Probiotics have inherent advantages in mycotoxin bio-control. In recent decades, various kinds of probiotic strains have been used for ZEN removal through degradation or biotransformation [16,17,18]. *Candida parapsilosis* can transform ZEN into the less toxic β-zearalenol and ZEN-14,16-diglucosid β-zearalenol [16]; *Candida utilis* and *Saccharomyces cerevisiae* remove ZEN by adsorption [17]. *Bacillus subtilis* Y816 can remove more than 95% of ZEN (20 mg/L) within 24 h by converting ZEN into ZEN-14-phosphate through novel phosphorylated binding [18]. Lactic acid bacteria (LAB), as natural microorganisms in intestinal flora, are ideal ZEN decontamination candidates. Moreover, most LAB have a prominently hydrophobic cell surface and abundant metabolic products (e.g., acetic acid, phenyllactic acid, and hydroxyphenyllactic acid) or enzymes (e.g., esterases), which prevent toxicity of the mycotoxin [5,19,20]. Zhang et al. isolated 27 *Lactobacillus plantarum* strains isolated from traditional fermented food, and the maximum ZEN removal rate reached 47.8% in 48 h [3]. Mokoena et al. reported that 68% to 75% of ZEN in mycotoxin-spiked maize meal products was reduced after the mixed culture of *Streptococcus lactis* and *L. delbrueckii* underwent fermentation for 4 days [21].

In the present study, the pickle-derived LAB with ZEN removal potential were isolated and identified, potent ZEN removal strains were selected, and the ZEN removal conditions were optimized. On this basis, the removal mechanisms of ZEN mycotoxin, both absorption and biotransformation, were studied through metabolite analysis, biochemical treatment analysis, volatile component analysis, Fourier-transform infrared spectroscopy (FTIR), and scanning electron microscopy (SEM).

## 2. Materials and Methods

### 2.1. Materials

Zearalenone (ZEN), α-zearalenol (α-ZOL), and β-zearalenol (β-ZOL) standards were purchased from Sigma-Aldrich (St. Louis, MO, USA) and stored at −20 °C. Stock solutions of ZEN (1 mg/L), α-ZOL (1 mg/L), and β-ZOL (1 mg/L) were prepared by dissolving them in 100% methanol. The standard solutions for high-performance liquid chromatography (HPLC) calibration were prepared by diluting the stock solution with a methanol/water (50/50, *v*/*v*) mixture. Other chemicals such as urea, sodium dodecyl sulfate (SDS), m-periodate, ploymyxin B, protease E, and lipase were purchased from Solarbio Science & Technology Co., Ltd. (Beijing, China). The Lactobacillus biochemical identification Kit and De Man Rogosa and Sharpe (MRS) medium were provided by Huankai Microbial Science & Technology Co., Ltd. (Guangzhou, China). All the other chemicals used in this experiment were of chromatographic or analytical grade.

### 2.2. Isolation and Purification of LAB from Pickles

Pickle samples were collected from Sichuan, China., In accordance with Rao et al. [22], samples were serial decimal diluted using 0.01 M phosphate buffered saline (PBS, pH 7.2), and the sample dilution was continuously streak-cultured on MRS agar plates at least 5 times to obtain a purified and single clone. In total, 87 single colonies were identified and transferred into tubes containing MRS broth, followed by incubation under anaerobic conditions at 37 °C for 48 h, then stored at −80 °C in MRS broth supplemented with 30% glycerol.

### 2.3. Strain Screening and Identification

To screen LAB with ZEN removal ability, each single clone was inoculated separately in MRS broth medium containing 5 mg/L ZEN and incubated at 37 °C for 48 h. After incubation, the cell suspensions were centrifuged at 10,000 rpm for 10 min at 4 °C. The supernatant was mixed with an equal volume of ethyl acetate, and then centrifuged at 8000 rpm for 2 min to collect the ethyl acetate phase. The collected organic solvent was evaporated using a water bath at 40 °C, the residue was dissolved in a methanol/water (50/50, *v*/*v*) mixture, and then filtered through a 0.22 µm filter before the HPLC analysis of ZEN [3,10].

The ZEN removal ability was calculated as

Dr = (1 − C_t_/C_c_) × 100%

where Dr represents the ZEN removal rate and C_t_ and C_c_ are the ZEN concentrations of the treatment and control, respectively. The control is the initial stage of strain cultivation.

The LAB with high ZEN removal ability (>50%) was identified using the Lactobacillus biochemical identification Kit following the manufacturer’s instructions. The 16S rDNA partial gene sequence was amplified using the universal primers 27F (5′-AGAGTTTGATCMTGGCTCAG-3′) and 1492R (5′-CGGTTACCTTGTTACGACTT-3′). Homologous analysis of the partial gene sequence of the isolate was performed online using NCBI BLAST software (http://blast.ncbi.nlm.nih.gov, the access date 11 November 2021).

### 2.4. Quantification of ZEN and Its Metabolites

The LCMS-8060 system (Shimadzu, Japan) was employed to detect the residual ZEN level. It was equipped with a triple quadrupole MS analyzer and an electrospray ionization (ESI) interface. Separation was achieved using HPLC, using a Shimadzu Shim-pack XR-ODS III (75 mm × 2.0 mm × 1.6 μm) column maintained at 40 °C [2]. The injection volume was 2 μL. The mobile phases were 5 mM ammonium formate (A) and methanol (B) working in isocratic mode at 0.3 mL/min. The elution gradient started at 10% B and increased linearly to 100% B after 3 min, and then held at 100% B for 2 min. Then, gradient washing returned to a 10% B within 1 min, and then held at 10% B for 2 min before the next injection. The MS detection was measured under multiple reaction monitoring (MRM). Recorded retention times for ZEN, α-ZOL, and β-ZOL were 3.86 min, 3.57 min, and 3.71 min, respectively.

### 2.5. Optimization of LAB Incubation Conditions for ZEN Removal

To improve the ZEN removal ability of LAB, the incubation conditions of LAB were optimized. The ZEN removal ability of the top 5 strains was optimized under the following conditions: initial substrate concentration (1, 5, 10, and 20 mg/L), incubation temperature (28, 37, and 42 °C), medium pH (2.0, 4.0, and 6.0) and incubation time (6, 12, 24, 36, and 48 h). The initial concentrations of LAB were 3.39 McFarland (10.17 × 10^8^ CFU/mL, *L. plantarum* 22), 3.27 McFarland (10.38 × 10^8^ CFU/mL, *L. plantarum* 37), 3.83 McFarland (11.49 × 10^8^ CFU/mL, *L. plantarum* 47), 3.46 McFarland (10.38 × 10^8^ CFU/mL, *L. paracasei* 85) and 3.83 McFarland (11.49 × 10^8^ CFU/mL, *L. buchneri* 93). When one factor was varied, the others were fixed. The same procedure was used for MRS medium without LAB and ZEN as a negative control, and LAB-free MRS medium contaminated with ZEN was used as a positive control. The obtained samples were analyzed by HPLC to determine the concentration of ZEN as described above.

### 2.6. ZEN Removal Abilities of Whole Cell Culture, Cell-Free Suspension, Cell Pellets, and Inactivated Cell Pellets

In order to clarify the main components of LAB that affect the ZEN removal process, the ZEN removal abilities of whole cell culture, cell-free suspension, cell pellets, and inactivated cell pellets were investigated. The whole cell culture of LAB was collected and centrifuged (10,000 rpm, 10 min, 4 °C), filtered to prepare a cell-free suspension, and the precipitate was washed twice with 10 mL of sterile PBS and suspended in sterile PBS to obtain cell pellets. Another 10 mL of LAB culture was sterilized at 121 °C for 20 min to collect inactivated cell pellets Then, ZEN (5 mg/L) was added into solutions of whole cell culture, cell-free suspension, cell pellets, and inactivated cell pellets. Sterile PBS containing 5 mg/L of ZEN was set as a control. These suspensions were incubated at 37 °C for 4 h, and then the ZEN levels were analyzed.

### 2.7. ZEN Metabolization by L. paracasei 85 and L. buchneri 93

The biotransformation of ZEN was investigated due to ZEN being reduced through both the formation of a ZEN-bacteria complex and the generation of metabolites such as α-ZOL and β-ZOL. After 48 h of LAB incubation with ZEN, the mixture was transferred to a sterile tube and centrifuged. The obtained supernatant and precipitate were extracted twice with equal volumes of chloroform. The obtained chloroform phases were combined and evaporated to dryness, following dissolution using acetonitrile. The obtained samples were then mixed in a vortex and placed in an ultrasonic bath (30 min, 40 °C) [2], then, the levels of ZEN, α-ZOL, and β-ZOL were analyzed.

### 2.8. Volatile Organic Compounds Analysis

The profile of volatile organic compounds (VOCs) produced by *L. paracasei* 85 and *L. buchneri* 93 were determined using an Agilent 7890bsystem (Santa Clara, CA, USA) coupled with Agilent 5977 Inert XL MSD apparatus. The system was equipped with a fused-silica capillary column coated with a polar stationary phase (OMEGAWAX™250, 30 m × 0.25 mm × 0.25 μm, Supelco, Bellefonte, PA, USA). The bacterial culture with or without ZEN treatment was inoculated with Tryptic Soy Agar (TSA) medium (Sigma-Aldrich, Steinheim, Germany). The vials were placed at an angle into the incubator. The VOCs were extracted with 50 μm polydimethylsiloxane (PDMS)/divinylbenzene (DVB) fiber (Supelco, Bellefonte, PA, USA) at 37 °C for 50 min. The absorbed molecules were desorbed into the injection port for 5 min at 250 °C. VOCs were separated with the following program: held 40 °C for 5 min, heated at 5 °C/min to 185 °C, held for 1 min, heated at 5 °C/min to 200 °C, held for 10 min, and then heated at 10 °C/min to 240 °C. Helium was used as the carrier gas. The MS interface and the ion source were maintained at 250 and 230 °C, respectively. Acquisition was performed in electron impact mode (70 eV) with the mass range of 30–300 *m*/*z*. Compounds were identified by matching mass spectra with the NIST14.1 library [2]. The analyses for each sample were carried out in triplicate.

### 2.9. Chemical and Enzymatic Treatment

To investigate the possible components of the bacterial cell which were involved in ZEN removal, the ZEN removal ability of LAB was detected after chemical and enzymatic treatments as previously described by Adunphatcharaphon et al. [23] Briefly, *L. paracasei* 85 and *L. plantarum* 93 were activated in MRS medium, and then the active cell suspensions were centrifuged as described above. The collected pellets were washed twice with PBS buffer and then reacted with chemical or enzymatic reagents. Treatment of the pellets was performed in the following process: 8 M urea (37 °C, 1 h), 0.1 M sodium dodecyl sulfate (SDS) (37 °C, 1 h), 10 mg/mL m-periodate (37 °C, 2 h), 10 mg/L ploymyxin B (37 °C, 4 h), 0.5 mg/mL pronase E (37 °C, 2 h), and 0.5 mg/mL lipase (37 °C, 2 h). After the reaction, the cell suspensions were centrifuged and suspended in PBS. Finally, the pellets were mixed with ZEN (5 mg/L) and incubated at 37 °C for 4 h to analyze ZEN level of suspension using LCMS-8060. Sterile PBS containing 5 mg/L of ZEN was set as a control.

### 2.10. Fourier-Transform Infrared Spectroscopy (FTIR) Analysis

Activated bacteria were incubated with or without ZEN (5 mg/L) at 37 °C. After 48 h of incubation, cells were washed twice and resuspended with PBS buffer (pH 7.2). Subsequently, 10 µL of the obtained suspension was pressed into the center of an IR transparent ZnSe optical card, and vacuum-dried in a desiccator in the presence of anhydrous silica. The infrared spectrum was recorded in the range of 400–4000 cm^−1^ using the Nicolet 6700 spectrometer (Thermo Electron Corporation, Madison, WI, USA). Spectra were recorded at a 1 cm^−1^ spectral resolution, and each spectrum was achieved by co-adding 50 scans.

### 2.11. Scanning Electron Microscopy (SEM) Analysis

For SEM analysis, LAB were exposed to ZEN (5 mg/L) and incubated at 37 °C for 48 h, separately, along with two strains without ZEN as controls. After incubation, MRS broth medium was removed and cell precipitations were washed three times with 0.1 M PBS. In accordance with Deepthi et al. [24], samples were fixed with 2.5% (*v*/*v*) glutaraldehyde overnight at room temperature, then rinsed three times with 0.1 M sodium phosphate buffer and dehydrated in ethanol solutions with increasing gradients (50%, 70%, 80%, 90% and 100%). Subsequently, the samples were air dried, mounted on an aluminum stub using double-sided carbon tape, sputter-coated with gold and observed using S3400N SEM (Hitachi, Japan) at 10,000× magnification.

### 2.12. Statistical Analysis

All the experiments were performed in triplicate, and the results are expressed as mean ± standard deviation (SD). Statistical analysis was carried out using SPSS version 17.0, and significant differences were assessed using the one-way ANOVA test (*p* < 0.05). The graphs were drawn using Graph Pad Prism version 7 (GraphPad Software Inc., San Diego, CA, USA).

## 3. Results

### 3.1. Screening and Identification of LAB for ZEN Removal

**Figure 1:**
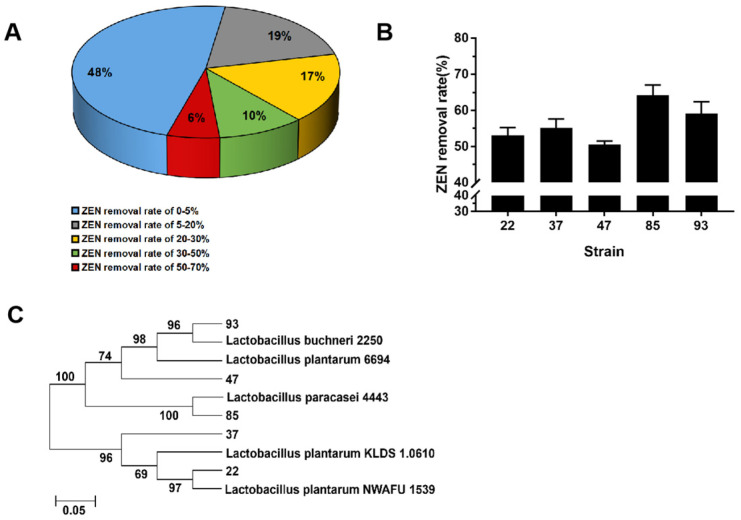
Strain screening for a high ZEN removal ability. (**A**) Distribution characteristics of the LAB derived from pickles (87 strains in total) according to the ZEN removal rate; (**B**) ZEN removal rate of the top 5 strains; (**C**) Phylogenetic relationship of the top 5 strains constructed by 16S rDNA sequences from evolutionary distances.

Among these selected 87 pickle-derived LAB strains, 45 strains had a ZEN removal ability of more than 5% (Figure 1A), of which 5 strains had an excellent ZEN removal ability (>50%). The No. 85 strain exhibited the highest ZEN removal ability (68.4%), followed by strains No. 93 (59.2%), No. 37 (57.4%), No. 22 (53.7%), and No. 47 (50.6%) (Figure 1B). The five strains with high ZEN removal ability had typical characteristics of LAB according to the carbohydrate reaction (Table 1), which was taxonomically consistent with the results of gene sequencing analysis. They were identified as *Lactobacillus plantarum* 22, *L. plantarum* 37, *L. plantarum* 47, *L. paracasei* 85, and *L. buchneri* 93.

### 3.2. Condition Optimization of LAB Incubation to Promote the ZEN Removal Potential

**Figure 2:**
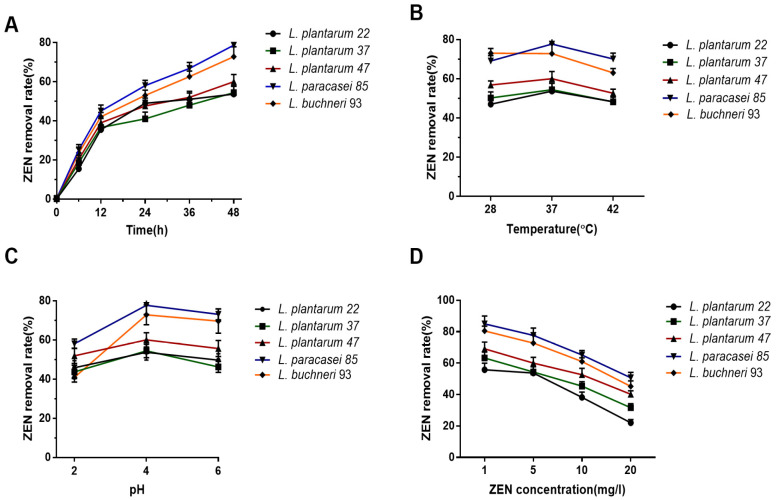
Optimization conditions of LAB incubation to promote the ZEN removal potential. (**A**) The effect of incubation time (6, 12, 24, 36, and 48 h) on the ZEN removal ability. (**B**) The effect of incubation temperature (28, 37, and 42 °C) on the ZEN removal ability. (**C**) The effect of pH media (2.0, 4.0, and 6.0) on the ZEN removal ability. (**D**) The effect of ZEN concentration (1, 5, 10, and 20 mg/L) on the ZEN removal ability.

As shown in Figure 2A, the optimal incubation time of ZEN removal was 48 h, at which the highest removal rates of *L. paracasei* 85 and *L. buchneri* 93 reached 77.7% and 72.8%, respectively. Although *L. buchneri* 93 exhibited a potent clearance ability against ZEN at 28 °C, all the other four LAB strains exhibited the highest ZEN removal ability at 37 °C (Figure 2B). Moreover, the pH sensitivity of LAB to ZEN removal ability presented that the maximum ZEN removal rate of these five strains was observed at pH 4.0; in other words, pH 4.0 is the optimum pH for ZEN removal (Figure 2C). Finally, the ZEN removal ability was found to be negatively correlated with ZEN concentration. As shown in Figure 2D, the highest ZEN removal rates (≥55.8%) were observed at ZEN 1mg/L in all the tested LAB, when the concentration of ZEN increased to 5 mg/L the ZEN removal rates (≥53.7%) showed a mild decline. Considering the ZEN content in contaminated feed products and the ZEN removal rate, 5 mg/L of ZEN was selected as the concentration in the following experiments. Overall, the results showed that the optimal conditions for ZEN removal were LAB incubated with ZEN (5 mg/L) for 48 h in MRS medium (pH 4.0, 37 °C).

### 3.3. Adsorption and Biodegradation Participated in ZEN Removal

In order to further reveal the relevant mechanism of the ZEN removal of LAB, ZEN was incubated with whole cell culture, cell-free suspension, cell pellet, and heat-treated inactivated cell pellet. As shown in Table 2, bacteria pellets played the main role in ZEN removal as opposed to the cell-free suspension. Specifically, the ZEN removal ability of *L. paracasei* 85 cell-free suspension reached 49.0%, which was stronger than those of the other strains in cell-free suspension. The removal ability of LAB cells increased after the heat treatment of the ZEN toxin. The results could preliminarily indicate that both absorption and biotransformation are associated with ZEN removal of the LAB. Since *L. paracasei* 85 and *L. buchneri* 93 exhibited excellent ZEN removal ability (>70%), which was significantly higher than the other three strains, *L. paracasei* 85 and *L. buchneri* 93 were selected as the target strains in subsequent analyses.

### 3.4. Reduction in ZEN Toxicity through LAB Metabolization

**Figure 3:**
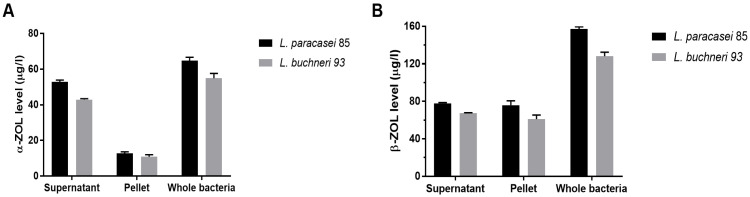
The levels of α-ZOL and β-ZOL in the supernatant, pellet, and whole bacteria of *L. paracasei* 85 and *L. buchneri* 93 after ZEN co-incubation. (**A**) The levels of α-ZOL, (**B**) The levels of β-ZOL.

The structures of ZEN, α-ZOL, and β-ZOL were similar to natural estrogen, therefore, they could competitively bind to the estrogen receptors, resulting in acute reproductive and genetic toxicity. α-ZOL especially, showed higher estrogenicity and competitive binding to estrogen receptors than parent ZEN, the relative toxicity was in the order α-ZOL > ZEN > β-ZOL [4]. The concentrations of ZEN derivatives are depicted in Figure 3. In the supernatant, there were higher α-ZOL levels (52.7 µg/L and 43.3 µg/L) than bacteria pellets (12.8 µg/L and 10.9 µg/L) in these two bacteria. However, in the pellets, the levels of β-ZOL (76.1 µg/L and 60.9 µg/L) were higher than the supernatant after ZEN treatments. In total, β-ZOL (157.7 µg/L and 128.2 µg/L) metabolized from ZEN were more than two-fold higher than α-ZOL (65.6 µg/L and 54.2 µg/L) in both whole bacteria of *L. paracasei* 85 and *L. buchneri* 93. Thus, the cell pellet and cell supernatant participated in the degradation of ZEN.

### 3.5. Influence of ZEN Degradation on LAB Volatile Organic Compounds (VOCs) Profile

**Figure 4:**
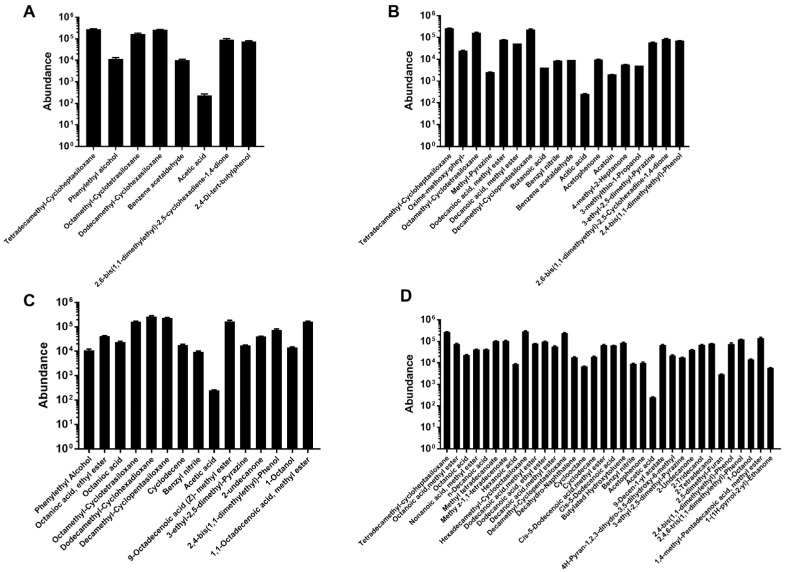
The profile of volatile organic compounds (VOCs). (**A**,**B**) The profile of VOCs of *L. paracasei* 85 without or with ZEN treatment. (**C**,**D**) The profile of VOCs of *L. buchneri* 93 without or with ZEN treatment.

LAB would produce various VOCs during fermentation, and some of them have essential effects on ZEN decontamination [25]. ZEN treatment significantly stimulated the production of VOCs in both of these two strains. Figure 4A displays all the VOCs extracted from *L. paracasei* 85, eight compounds of bacterial origin were determined, including acetic acid, tetradecamethyl-cycloheptasiloxane, benzeneacetaldehyde, phenylethyl alcohol, octamethyl-cyclotetrasiloxane, octadecamethyl-cyclononasiloxane, and 2,6-bis(1,1-dimethylethyl)-2,5-cyclohexadiene-1,4-dione and 2,4-di-tert-butylphenol. Another 12 VOCs containing butanoic acid extracted from *L. paracasei* 85 existed in the ZEN supplementation group (Figure 4B). However, phenylethyl alcohol and 2,4-di-tert-butylphenol were not detected after ZEN treatment. As for *L. buchneri* 93, 15 types of VOCs were extracted, and after incubation with ZEN, the different types of VOCs increased to 34 (Figure 4D). Except for 8 collective VOCs proceeded by the *L. buchneri* 93 alone group, there are 26 different volatile metabolites only secreted by *L. buchneri* 93 with ZEN, including nonanoic acid methyl ester, n-decanoic acid, hexanoic acid, methyl tetradecanoate, methy z-1,1-tetradecenoate, cis-5-dodecenoic acid methyl ester, etc. The different secondary metabolites secreted by *L. paracasei* 85 and *L. buchneri* 93 included organic acid ketones, hydrocarbons, and esters, which may participate in ZEN degradation.

### 3.6. The Effect of Chemical and Enzymatic Treatments on ZEN Removal

**Figure 5:**
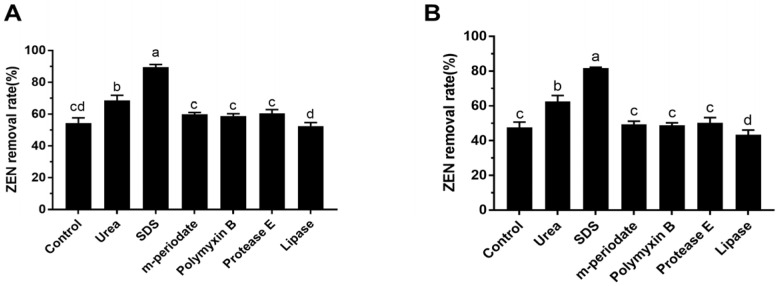
Effect of chemical and enzymatic treatments on the ZEN removal rate of pickle-derived LAB. (**A**) *L. paracasei* 85; (**B**) *L. buchneri* 93. a, b, c, and d: bars not sharing the same letters are significantly different (*p* < 0.05) from each other.

To investigate the possible components of the bacterial cell which were involved in the ZEN decontamination, these two LAB strains were treated with six different chemical and enzymatic ingredients, with the ZEN removal rates shown in Figure 5. The ZEN removal abilities were significantly inhibited after m-periodate (10 mg/mL) exposed to *L. paracasei* 85 and *L. buchneri* 93 for 2 h, which were decreased to 56.4% and 50.1%, respectively. SDS treatment significantly increased the ZEN removal abilities of *L. paracasei* 85 and reached 86.3% (Figure 5A), whereas this could not be observed in *L. buchneri* 93 (Figure 5B). Moreover, the protein destabilization induced by urea inhibited the capacity of *L. buchneri* 93 to effectively remove ZEN (*p* < 0.01). The lipase partially decreased the ZEN removal rate of *L. paracasei* 85 (*p* < 0.05), whereas no significant effect on ZEN removal by *L. buchneri* 93 was observed.

### 3.7. FTIR Analysis of LAB after ZEN Incubation

**Figure 6:**
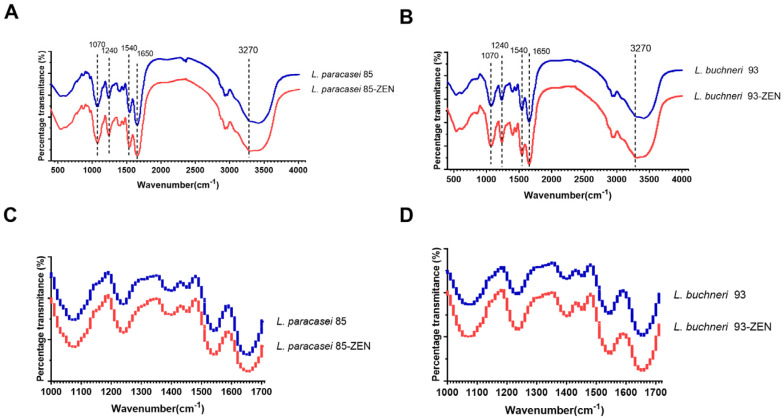
FTIR spectra of *L. paracasei* 85 and *L. buchneri* 93 with or without ZEN incubation. The FTIR spectra of *L. paracasei* 85 in (**A**) (400–4000 cm^−1^) and (**B**) (1000–1700 cm^−1^). The FTIR spectra of *L. buchneri* 93 in (**C**) (400–4000 cm^−1^) and (**D**) (1000–1700 cm^−1^).

In order to estimate the potential functional groups and the possible adsorption sites involved in the ZEN removal of *L. paracasei* 85 and *L. buchneri* 93, the FTIR analysis was conducted. The FTIR spectra of *L. paracasei* 85 and *L. buchneri* 93 incubated with or without ZEN are shown in Figure 6. The peak vibrations of these two bacterial cells were similar. The registered spectra revealed the changes in peak shapes and areas of these two strains after incubation with ZEN. As Figure 6C,D shows, the analyses of the bacteria spectroscopic spectra were focused on vibrations for groups of bacterial amides (ν = 1000–1700 cm^−1^). On the obtained spectra, the signal at ν = 1540 cm^−1^ indicated the presence of phenyl ring vibrations [26]. The presence of the peak at ν = 1650 cm^−1^ probably corresponded to the carbonyl group of the ZEN ring. These results are consistent with previous findings, in that ZEN removal by *L. plantarum* BCC 47,723 did not completely destroy the original structure, and the main compound structures of the cells were retained after binding to the cell wall [23]. Nevertheless, signals appeared in the range of 3180–3410 cm^−1^ were attributed to the O-H and N-H stretching vibrations of the amide group region characteristic for proteins [27]. Spectral bands observed at ν = 1070 cm^−1^ and 1240 cm^−1^ derived from carbon-nitrogen stretching vibrations, with the intensities of corresponding areas moderately increasing after ZEN treatment.

### 3.8. The Damage of Cell Morphology

**Figure 7:**
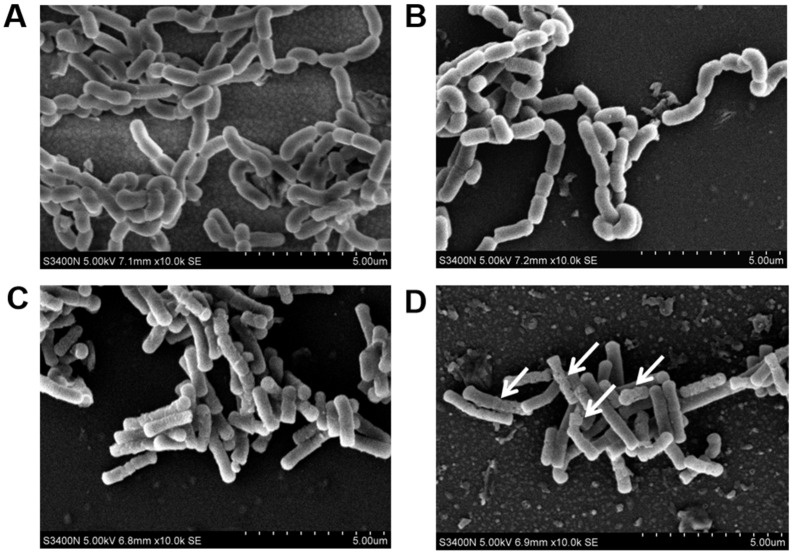
SEM photographs of bacterial cells at a magnification of 10,000×: (**A**) *L. paracasei* 85 without ZEN treatment; (**B**) *L. paracasei* 85 with ZEN treatment; (**C**) *L. buchneri* 93 without ZEN treatment; (**D**) *L. buchneri* 93 with ZEN treatment (the damage positions are marked with white arrows).

SEM was used to observe the morphology of LAB after ZEN treatment. SEM photographs (10,000×) of *L. paracasei* 85 and *L. buchneri* 93 with or without ZEN treatment are depicted in Figure 7, showing the different morphologies of *L. paracasei* 85 and *L. buchneri* 93. The *L. paracasei* 85 bacterium was a short or long, rod-shaped with rounded ends bacterium, and arrangement mode was short- or long-chain. While in *L. buchneri* 93, cells occurred as single or short chains of rods with rounded ends. There were no differences observed between the treated and untreated groups of *L. paracasei* 85 cell morphology (Figure 7A,B), thus ZEN binding to *L. paracasei* 85 made no effect on bacterial morphology integrity. The morphology of the *L. buchneri* 93 cell wall presented as more wrinkled and uneven (marked with white arrows) after the adsorption process, i.e., cell wall structure damage may have been caused by ZEN degradation.

## 4. Discussion

Fusariotoxin ZEN, a potent disrupter of the reproductive and immune system, is widely present widely in cereals and animal feed worldwide [28]. Previous studies have demonstrated that ZEN can be removed or biotransformed by various bacteria (i.e., *Lactobacillus*), yeasts, and fungi through converting ZEN to α- and β-zearalenol [29,30,31]. However, few studies have explained the related mechanisms of ZEN decontamination by LAB. LAB have various beneficial effects on health, such as anticancer, immunomodulatory, antidiarrheal, antioxidant and antimicrobial activities [32]. In the present study, 87 LAB strains were isolated from Sichuan pickles, 27 of which (10^9^ CFU/mL) demonstrated ZEN (5 mg/L) removal abilities ranging from 20% to 80%. The top five strains with a ZEN removal ability over 50% were further characterized as *L. plantarum* (3), *L. paracasei* (1) and *L. buchneri* (1); the maximum ZEN removal ability reached 68.4% for *L. paracasei* 83. The highest ZEN (1 mg/L) removal abilities of *L. paracasei* 85 and *L. buchneri* 93 were increased to 85.2% and 80.5% under the optimized conditions of 37 °C and pH 4.0 for 48 h. Hence, ZEN was removed by LAB in a concentration- and species-dependent manner. The removal abilities of LAB in the study are higher than in Čvek’s study, in which 17 out of 33 plant-derived LAB strains presented a ZEN (0.2 µg/L) removal ability ranging from 0.5% to 23% [33]. Moreover, heat treatment significantly enhanced the removal ability of pickle-derived LAB, which was consistent with the previous findings that the ZEN removal capacity of *L. plantarum* BCC47723 was significantly enhanced under 62 °C for 0.5 h [23]. Moreover, heat-treated yeast also showed a higher absorption of OTA in comparison with live cells [34]. This could be due to the fact that heat stress causes some new binding sites to be exposed and increases the affinity of bacteria binding to ZEN. In the present study, the pellets exhibited a higher ZEN removal ability than the cell-free supernatant, which means that most of the ZEN was adsorbed to the bacteria surface and taken into the cell; a minority of ZEN was biotransformed by the metabolites in cell supernatant. Therefore, both the cell pellets and supernatant participated in ZEN removal.

The chemical structure of ZEN is similar to natural estrogens and can bind to estrogen receptors to disturb the natural hormonal balance in animals [4]. α-ZOL and β-ZOL are key metabolites of ZEN biodegradation by microorganisms. The relative estrogenicity of α-ZOL is nearly 500 times stronger than that of ZEN, but that of β-ZOL is 16 times weaker than that of ZEN. Therefore, the transformation of ZEN to α-ZOL can enhance the toxicity of ZEN, and degradation of ZEN to β-ZOL can reduce the toxicity of ZEN [35]. In this study, α-ZOL was mainly produced in cell supernatant, and β-ZOL was mainly produced in cell precipitation. The levels of β-ZOL produced by LAB were more than two-fold higher than α-ZOL in both of these two strains, which showed that the metabolic process can partially decrease the toxicity of ZEN, and the supernatant also demonstrated detoxification abilities. When ZEN was degraded to β-ZOL, it decreased the affinity to estrogen receptors compared with ZEN and α-ZOL, thus attenuating the health threat. The strains *L. paracasei* 85 and *L. buchneri* 93 partially impaired the toxicity of ZEN by biotransforming ZEN to β-ZOL.

LAB are Gram-positive microorganisms with cell walls, the components of which include peptidoglycan, teichoic acids, polysaccharides, and proteins; differences in the structure and components of the cell wall play an important role in ZEN removal. According to the profile of VOCs, acetic acid is the only collective VOC in *L. paracasei* 85 and *L. buchneri* 93, which could be related to the fermentation characteristic metabolite of LAB [36]. The amounts of octanoic acid methyl ester were significantly increased after ZEN treatment in *L. buchneri* 93, whereas those of phenylethyl alcohol, octamethyl-cyclotetrasiloxane, dodecamethyl-cyclohexadioxane, 9-octadecenoic acid (Z)-methyl ester, 3-ethyl-2,5-dimethyl- pyrazine, and 1,1-octadecenoic acid methyl ester were decreased. Therefore, the supplementation of ZEN affected the fermentation characteristic of LAB by altering the profile of VOCs. According to Chen’s study, the fermentation characteristics of ZEN-contaminated maize were ameliorated by the *Bacillus* B2 strain. ZEN increased the levels of bacteria numbers, lactic acid, acetic acid, total volatile fatty acids, and ammonia nitrogen [37]. In addition, m-Periodate treatment significantly decreased the ZEN removal rates in *L. paracasei* 85 and *L. buchneri* 93 (Figure 5). m-Periodate oxidized cis-OH groups to aldehydes and carbon acid groups of the exopolysaccharide, which was essential for ZEN binding [38]. SDS can insert into the hydrophobic interior of the protein to break down and disrupt the secondary forces of proteins. Protein unfolding induced by SDS significantly increased the ZEN removal abilities of *L. paracasei* 85. In contrast, the lipase partially decreased the ZEN removal rate of *L. paracasei* 85 (*p* < 0.05), because it is an enzyme with various catalytic abilities, including hydrolysis, alcoholysis, and esterification. Regarding *L. buchneri* 93, the ZEN removal ability was also inhibited by urea, through forming hydrogen bonds to the peptide group and impairing the stability of secondary and tertiary structures of protein [39]. When protein was broken down into the unfolded state, foreign objects can easily cross into the hydrophobic core of the wall and lead to a decrease in ZEN bounding ability. Overall, the bacterial cell surface elements, such as exopolysaccharides, esters, and proteins, played key roles in the ZEN removal.

The potential functional groups and the possible sites related the adsorption of ZEN for *L. paracasei* 85 and *L. buchneri* 93 were further identified by FTIR analysis. According to the obtained spectra, the peak intensity at 1070 cm^−1^ was increased after incubation with ZEN, indicating that strong asymmetrical stretching occurred. Similarly, a slight change in the percentage transmittance at 1240 cm^−1^ was also observed, further confirming that exopolysaccharides were involved in ZEN adsorption [2]. In addition, SEM graphs showed that *L. buchneri* 93 presented a more wrinkled and uneven surface when incubated with ZEN, suggesting that the transformation of the cell surface structure might occur coupled with ZEN removal. Although both *L. paracasei* 85 and *L. buchneri* 93 were equal in ZEN clearance ability, they have different levels of sensitivity to ZEN removal, and *L. paracasei* 85 has a stronger tolerance than *L. buchneri* 93.

## 5. Conclusions

There were 87 strains of LAB isolated from Sichuan pickles, with ZEN removal abilities ranging from 0% to 68.4%. The top five strains with potent ZEN removal abilities were identified as *L. plantarum* 22, *L. plantarum* 37, *L. plantarum* 47, *L. paracasei* 85, and *L. buchneri* 93. The strongest removal abilities of *L. paracasei* 85 and *L. buchneri* 93 reached 77.7% and 72.8% under the condition of 37 °C pH 4.0 to ZEN (5 mg/L). The degradation of ZEN occurred to both α-ZOL and β-ZOL; β-ZOL was a predominant metabolite in both the cell-free supernatant and pellet of *L. paracasei* 85 and *L. buchneri* 93. Various organic acids, alcohols, and esters were changed during ZEN removal, and the exopolysaccharides, proteins, and lipids on the cell wall were combined with ZEN to increase the removal rate through hydrophobic interactions. Specifically, ZEN removal was coupled with cell morphology and structural damage in *L. buchneri* 93. In conclusion, *L. paracasei* 85 and *L. buchneri* 93 can serve as candidate strains for the removal of ZEN in feed and foodstuff during the storage and transportation process through both absorption and biotransformation.

## Figures and Tables

**Table 1:** Carbohydrate reactions of the top 5 LAB strains with high ZEN removal ability.

	22	37	47	85	93
Esculin	+	+	+	+	+
Cellobiose	+	+	+	+	+
Maltose	+	+	+	+	+
Mannitol	+	+	+	+	−
Salicin	+	+	+	+	+
Sorbierite	+	+	+	−	−
Sucrose	+	+	+	+	+
Raffinose	+	+	+	−	−
pH	3.30	3.41	3.34	3.45	2.82
Logarithmic period	6–9 h	9–12 h	6–8 h	4–6 h	8–12 h

+, positive; −, negative.

**Table 2:** ZEN removal abilities of whole cell culture, cell-free supernatant, cell pellet, and inactivated cell pellets of pickle-derived LAB.

Isolate	ZEN Removal Rate (%)			
	Whole Cell Culture	Cell-Free Supernatant	Cell Pellet	Inactivated Cell Pellet
*L. plantarum* 22	65.7 ± 2.4 ^Abc^	33.5 ± 0.7 ^Ccd^	46.7 ± 3.6 ^Bb^	70.2 ± 3.8 ^Ac^
*L. plantarum* 37	67.8 ± 4.4 ^Ab^	32.7 ± 2.1 ^Cd^	54.1 ± 2.9 ^Bb^	68.2 ± 2.0 ^Ab^
*L. plantarum* 47	60.6 ± 3.9 ^Ac^	36.9 ± 1.5 ^Cc^	50.4 ± 2.0 ^Bb^	65.6 ± 2.4 ^Abc^
*L. paracasei* 85	78.4 ± 2.2 ^Aa^	49.0 ± 3.2 ^Ca^	66.1 ± 3.0 ^Ba^	80.8 ± 4.3 ^Aa^
*L. buchneri* 93	73.0 ± 1.6 ^Aa^	43.1 ± 1.1 ^Cb^	60.9 ± 2.4 ^Ba^	76.4 ± 3.5 ^Aa^

Note: Different upper-case letters indicate a significant difference within the same line (*p* < 0.05). Different lower-case letters indicate a significant difference within the same column (*p* < 0.05).

## Data Availability

The data presented in this study are available on request from the corresponding author. The data are not publicly available due to privacy restrictions.
